# Clioquinol Synergistically Augments Rescue by Zinc Supplementation in a Mouse Model of Acrodermatitis Enteropathica

**DOI:** 10.1371/journal.pone.0072543

**Published:** 2013-08-28

**Authors:** Jim Geiser, Robert C. De Lisle, David Finkelstein, Paul A. Adlard, Ashley I. Bush, Glen K. Andrews

**Affiliations:** 1 Department of Biochemistry and Molecular Biology, University of Kansas Medical Center, Kansas City, Kansas, United States of America; 2 Department of Anatomy and Cell Biology, University of Kansas Medical Center, Kansas City, Kansas, United States of America; 3 The Florey Institute of Neuroscience and Mental Health and the University of Melbourne, Victoria, Australia; Faculdade de Medicina Dentária, Universidade do Porto, Portugal

## Abstract

**Background:**

Zinc deficiency due to poor nutrition or genetic mutations in zinc transporters is a global health problem and approaches to providing effective dietary zinc supplementation while avoiding potential toxic side effects are needed.

**Methods/Principal Findings:**

Conditional knockout of the intestinal zinc transporter *Zip4* (*Slc39a4*) in mice creates a model of the lethal human genetic disease acrodermatitis enteropathica (AE). This knockout leads to acute zinc deficiency resulting in rapid weight loss, disrupted intestine integrity and eventually lethality, and therefore provides a model system in which to examine novel approaches to zinc supplementation. We examined the efficacy of dietary clioquinol (CQ), a well characterized zinc chelator/ionophore, in rescuing the *Zip4* ^intest KO^ phenotype. By 8 days after initiation of the knockout neither dietary CQ nor zinc supplementation in the drinking water was found to be effective at improving this phenotype. In contrast, dietary CQ in conjunction with zinc supplementation was highly effective. Dietary CQ with zinc supplementation rapidly restored intestine stem cell division and differentiation of secretory and the absorptive cells. These changes were accompanied by rapid growth and dramatically increased longevity in the majority of mice, as well as the apparent restoration of the homeostasis of several essential metals in the liver.

**Conclusions:**

These studies suggest that oral CQ (or other 8-hydroxyquinolines) coupled with zinc supplementation could provide a facile approach toward treating zinc deficiency in humans by stimulating stem cell proliferation and differentiation of intestinal epithelial cells.

## Introduction

Zinc deficiency is a global health problem [1] and approaches for providing effective dietary zinc supplementation in developing countries are under active clinical investigation. Dietary zinc deficiency correlates with low birth weight and poor pregnancy outcome in humans [2]–[4], and has been associated with abnormal fetal development [5], diabetes and schizophrenia [6], decreased cognitive behavior and intellectual development [7], [8], and impaired host defense [9]. Zinc deficiency increases mortality and disease [10]. These effects reflect the remarkably diverse functions of the essential metal zinc which serves structural and/or catalytic roles in hundreds of peptides, including the zinc finger proteins [11]–[13]. However, zinc is also toxic when in excess and can attenuate copper and iron [14] uptake, even leading to mortality [15], [16] or memory impairment [17] or acute pancreatitis [18]–[20]. Therefore, the maintenance of zinc homeostasis is critical and approaches to facilitating the efficacy and reducing the toxicity of zinc supplementations are needed.

There are several causes of zinc deficiency in humans in addition to a lack of sufficient nutrition. Diets high in phytate, which is found in many plants, can reduce the bioavailability of ingested zinc due to chelation [21]. Individuals with compromised intestines, such as Crohn’s disease, diverticulitis and alcoholism, also absorb zinc poorly [22]. Childhood diarrheas, which can be fatal, are associated with zinc deficiency and infant mortality in some developing countries [1]. In addition, there are mutations in zinc transporters which cause zinc deficiency syndromes, such as transient neonatal zinc deficiency which results from the lack of sufficient zinc in the mother’s milk [23] and a form of Ehlers-Danlos syndrome which results from impaired development of bone, teeth and connective tissue [24]. A devastating, lethal genetic disease of zinc metabolism is acrodermatitis enteropathica (AE) which is caused by compromised absorption of dietary zinc [25], [26]. AE symptoms develop after birth in bottle fed infants or after weaning in breast fed infants and patients must be provided lifelong zinc supplementation or they suffer from a myriad of symptoms of severe zinc deficiency which eventually lead to death if not treated with zinc [27]. However, some patients are resistant to zinc supplementation [28] and as mentioned above excess zinc can affect the homeostasis of other essential metals.

We developed a conditional knockout mouse model of AE that recapitulates many aspects of the human disease and which provides a unique tool to study the progression and underlying causes of this disease as well as to study potential approaches to treating AE. In this model the loss-of-function of the zinc transporter ZIP4 (Slc39a4) specifically in the intestine rapidly causes zinc deficiency which is accompanied by a disruption of the integrity of the intestine and repression of the division of intestine stem cells and differentiation of their progeny [26]. These changes contribute to a rapid switch from anabolic to catabolic metabolism, tissue-specific dysregulation of other essential metals and death of the knockout mice. Providing zinc supplementation by including high concentrations of zinc in the drinking water could rescue these mice if given immediately after initiation of the *Zip4* knockout. However, the efficacy of zinc supplementation to rescue these mice, under these experimental conditions, diminishes during progression of the disease. Therefore, we examined whether a zinc ionophore might increase the efficacy of zinc uptake in these mice once the intestine is compromised.

A well-studied zinc chelator/ionophore is 5-chloro-7-iodo-8-hydroxyquinoline or clioquinol (CQ) [29], [30]. Almost 80 years ago CQ was first employed as an antibiotic in the treatment of parasitic infections and diarrhea. In those cases CQ may have functioned to compete for available zinc and copper thereby restricting growth of the pathogens. A related compound diiodohydroxyquinoline (diodoquin) and CQ were later used to treat patients with AE but their mechanism of action was obscure at that time [27], [31]–[33] as was the etiology of AE. These compounds were withdrawn from the market in the 1970s after the appearance of SMON (sub-acute myelo-optic neuropathy) in Japan and in some AE patients [34]. The preponderance of evidence suggests that this unique syndrome was not caused by CQ alone but was related to zinc deficiency and malnutrition [30], [34]. Later in the 70′s AE was recognized as a zinc deficiency disease [27] and it was understood that CQ was functioning as an ionophore for zinc [35], [36]. Thereafter, most AE patients were treated effectively with lifelong zinc supplementation but not with CQ. More recently CQ and related 8- hydroxyquinolines have reemerged as potential treatments for neurodegenerative disorders such as Alzheimer’s disease [29]. These compounds are thought to function, in this context, by chelating zinc and copper from lower affinity binding sites in Aβ plaques while acting as metallochaperones (ionophores) that can shuttle these metals to important cellular ligands rather than remove them from bioavailability [29]. However, it has also been suggested that the chelating properties of CQ can deplete copper and increase lethality in amyloid precursor protein (APP) transgenic mice [37]. CQ binds zinc with moderate affinity but it chelates copper with a 10-fold higher affinity and can also bind iron. The therapeutic value of these compounds in this context requires further investigation.

Given the well-studied pharmacokinetics and toxicology of CQ and the availability of many different 8-hydroxyquinolines which exhibit distinct biological activities [38], we examined whether CQ could reverse the effects of severe and extreme zinc deficiency in a mouse model of AE. We found that dietary CQ alone was ineffective in that regard, but the combination of dietary CQ and zinc supplementation acted synergistically and could reverse the effects of extreme zinc deficiency in most but not all of these mice. Dietary CQ and zinc supplementation rapidly stimulated intestine stem cell division leading to the restoration of intestine integrity. This was accompanied by rapid growth of the mice, restoration of the homeostasis of several essential metals and dramatically extended longevity. Therefore, oral CQ (or other 8-hydroxyquinolines) coupled with zinc supplementation, under the proper conditions, could be a highly effective approach toward the treatment of zinc deficiency in humans.

## Results

### Dietary CQ Acts Synergistically with Zinc Supplementation

**Figure 1:**
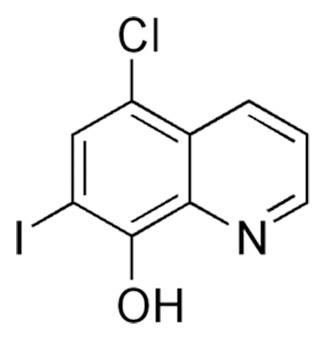
Structure of clioquinol. CQ is considered an intermediate affinity zinc chelator (Log conditional stability constant = 8.8; [67]) that also functions as a zinc ionophore. The ligand to metal stoichiometry is 1∶2 [67], [68]. Its mechanism of action and clinical uses have been recently reviewed [29], [30].

**Figure 2:**
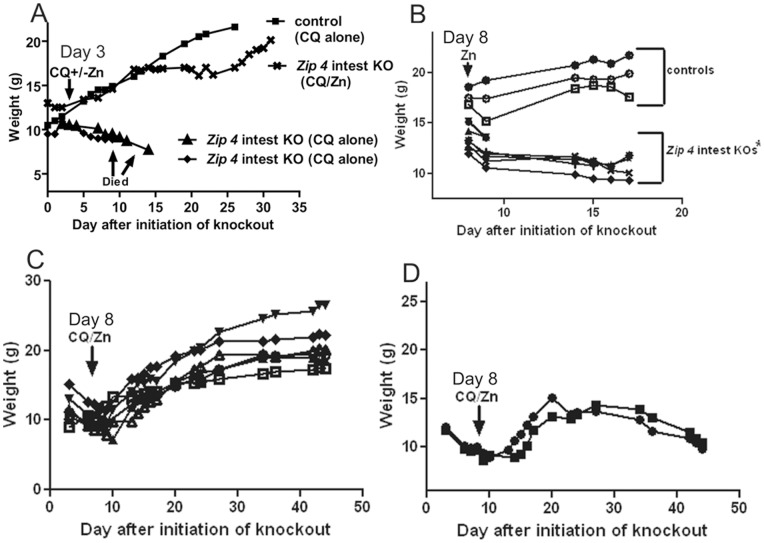
Dietary CQ with excess zinc in the drinking water (CQ/Zn) can rescue *Zip4* ^intest KO^ mice from the lethal effects of extreme zinc deficiency. Mice homozygous for the floxed *Zip4* gene and positive for the *vil-CreERT2* gene (***Zip4*** **intest KO**) and littermates homozygous for the floxed *Zip4* gene but negative for the *vil-CreERT2* gene (**control**) were injected for 3 consecutive days with tamoxifen beginning 2 to 3 weeks after weaning and their body weights were monitored thereafter. These mice were fed normal chow and provided access to deionized water after weaning and then fed chow containing CQ and/or provided water containing excess zinc beginning on the indicated days. (**A**) Mice were fed chow containing CQ (**CQ**) or CQ chow plus water containing excess zinc (**CQ/Zn**) beginning 3 days after initiation of the intestine knockout. Only representative mice are shown for the sake of clarity in the figure. (**B**) On d8 after initiation of the intestine knockout mice were provided with water containing excess zinc. (**C**) On d8 after initiation of the intestine knockout these mice were fed chow containing CQ and provided access to drinking water containing excess zinc. Among these mice 6/8 thrived while (**D**) 2 did not thrive and one appeared to be blind in one eye.

We previously reported that *Zip4* ^intest KO^ mice could be rescued from precipitous weight loss and death by giving them access to zinc in the drinking water (250 ppm ZnS0_4_) beginning on the day the knockout was initiated [26]. However, the efficacy of zinc supplementation, under these experimental conditions, rapidly diminishes if administration is delayed. For example, within 2 days after initiation of the knockout providing zinc supplementation does not reliably stimulate growth or significantly extend longevity in these mice (n = 9; data not shown). This observation prompted us to examine whether the zinc ionophore CQ (Fig. 1) could increase the efficacy of zinc supplementation to reverse the catabolic state in these mice and support long term survival and growth. Mouse chow containing CQ (250 ppm) in the presence or absence of zinc supplementation in drinking water was provided to *Zip4* ^intestKO^ mice starting on d3 after initiation of the knockout (Fig. 2A). Dietary CQ alone failed to rescue these mice and they were euthanized within 9 to 14 days after initiation of the knockout (n = 5). In sharp contrast, dietary CQ along with zinc supplementation in the drinking water (CQ/Zn) was highly effective (n = 5). Many of the *Zip4* ^intest KO^ mice treated in this manner thrived (3 out of 5 mice). They grew similarly to their control littermates and appeared healthy for the duration of this experiment (32 days). Two of these mice failed to gained weight but lived for the duration of this experiment before being euthanized (data not shown).

These observations were then extended to studies of *Zip4* ^intest KO^ mice on d8 after the knockout was initiated. This time was chosen because by then 22% of the knockout mice have been euthanized (21/96) and the surviving mice are extremely zinc deficient having lost a significant amount of bone, muscle mass [26]. Zinc supplementation in the drinking water failed to restore growth of these mice (Fig. 2B) and the majority were euthanized by d17 (12/14 mice). Two of these mice lingered to d32 but did not gain weight and were euthanized. Dietary CQ alone was also ineffective and 8/11 mice were euthanized by d14 and the other 3 weighed <9.4 g on d14 when the experiment was terminated and these mice were euthanized. In sharp contrast, dietary CQ/Zn (Fig. 2C) was highly effective at restoring growth and extending longevity in the majority of these mice (6/8 mice). Two of these mice did not thrive although their live span was extended to >45 days when the experiment was ended and these mice were euthanized (Fig. 2D). We repeated this latter experiment several times and among the mice that failed to thrive when fed CQ/Zn beginning on d6 or d8 we noted that 56% (9/16) appeared to be blind in one or both eyes.

**Figure 3:**
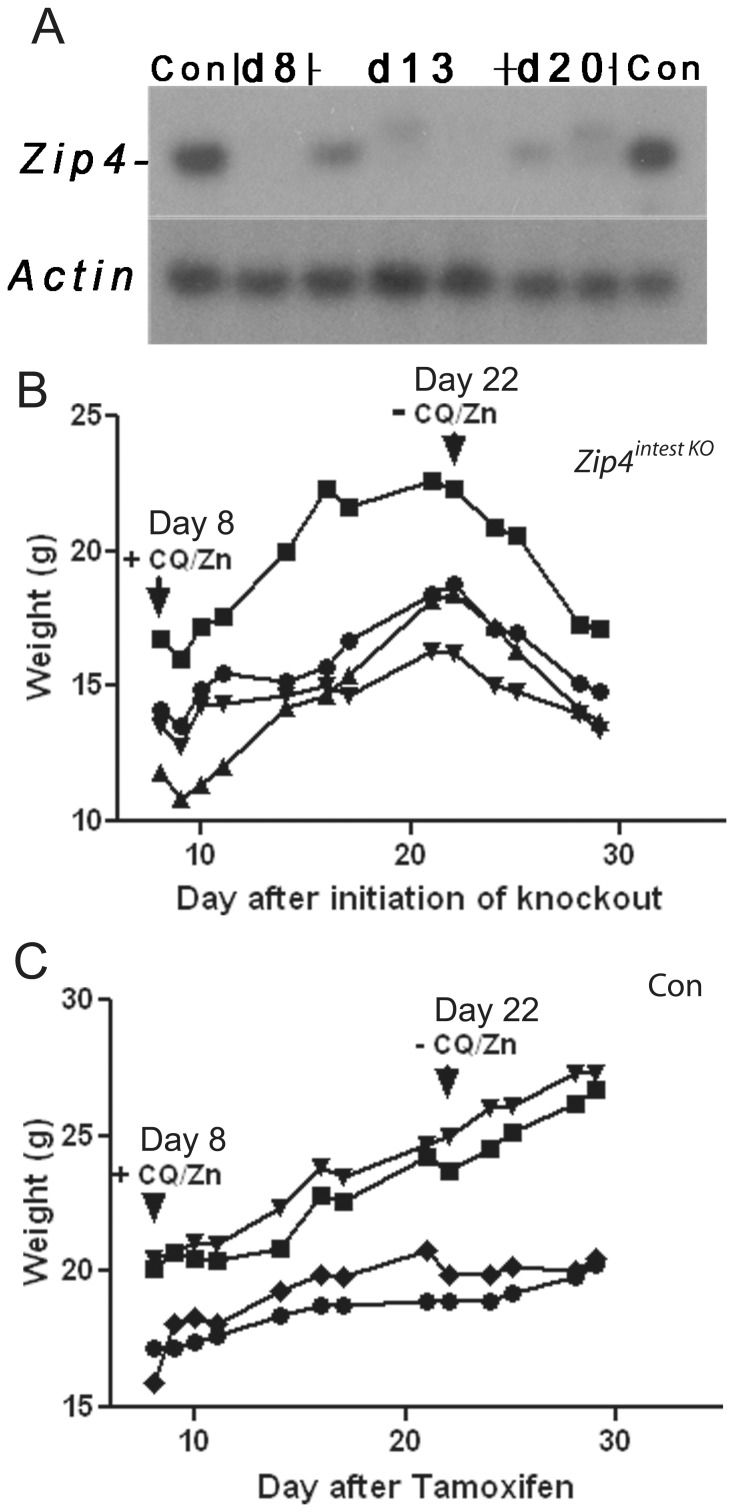
*Zip4* ^intest KO^ mice rescued by CQ/Zn remain dependent on CQ/Zn to survive. *Zip4* ^intest KO^ and control mice (n = 9 per group), as described in the legend to Figure 2, were fed normal chow and provided access to deionized water after weaning. On d8 after initiation of the knockout, mice were fed chow containing CQ and provided with water containing zinc **(+CQ/Zn)**. On d22 the CQ/Zn diet was replaced with normal chow and deionized water **(−CQ/Zn)** and the mice were monitored thereafter. (**A**) Northern blot analysis of intestinal *Zip4* mRNA from individual control mice (**Con**) and *Zip4* ^intest KO^ mice on **d8**, **d13** and **d20** (4 mice per group; one sample is shown for d8, three are shown for d13 and two are shown for d20). (**B**) Body weights of *Zip4* ^intest KO^ mice. (**C**) Body weights of control mice.

Our previous studies demonstrated that the *Zip4* gene remains knocked out for at least 4 weeks in these mice when zinc supplementation is continued from the time of initiation of the knockout [26]. However, incomplete efficiency of the *Zip4* gene knockout or regaining expression of *Zip4* due to stem cell proliferation could have contributed to the ability of these mice to recover when given the CQ/Zn diet. Therefore, we quantified *Zip4* mRNA abundance in the intestines of these mice (Fig 3A). For comparison the relative abundance of *Zip4* mRNA in control mice was also determined. Among 4 mice examined on d8, none had detectable *Zip4* mRNA. Transcripts from the knockout locus are detected but they do not co-migrate with authentic *Zip4* mRNA and are in low abundance (Fig. 3A: one d8 sample is shown). Surprisingly, *Zip4* mRNA transcripts were detected in 1 out of 4 mice examined on d13 and 1out of 4 mice on d20. Therefore, we determined whether *Zip4* ^intest KO^ mice rescued by CQ/Zn diet remain dependent on CQ/Zn. To that end, a group of mice (n = 8) were provided the CQ/Zn diet beginning on d8 and then the diet was replaced on d24 with normal chow and deionized water (Fig. 3B). All of these mice examined began to rapidly lose weight after removal of the CQ/Zn diet and 7 of those mice were euthanized within two weeks while one mouse lingered past two weeks and was euthanized. Control mice treated similarly continued to gain weight after removal of the CQ/Zn diet (Fig. 3C). Thus, the *Zip4* ^intest KO^ mice remained dependent on dietary CQ/Zn and recovery of expression of the *Zip4* gene does not account for the efficacy of this treatment.

### Dietary CQ with Zinc Supplementation Restores Intestine Stem Cell Division

**Figure 4:**
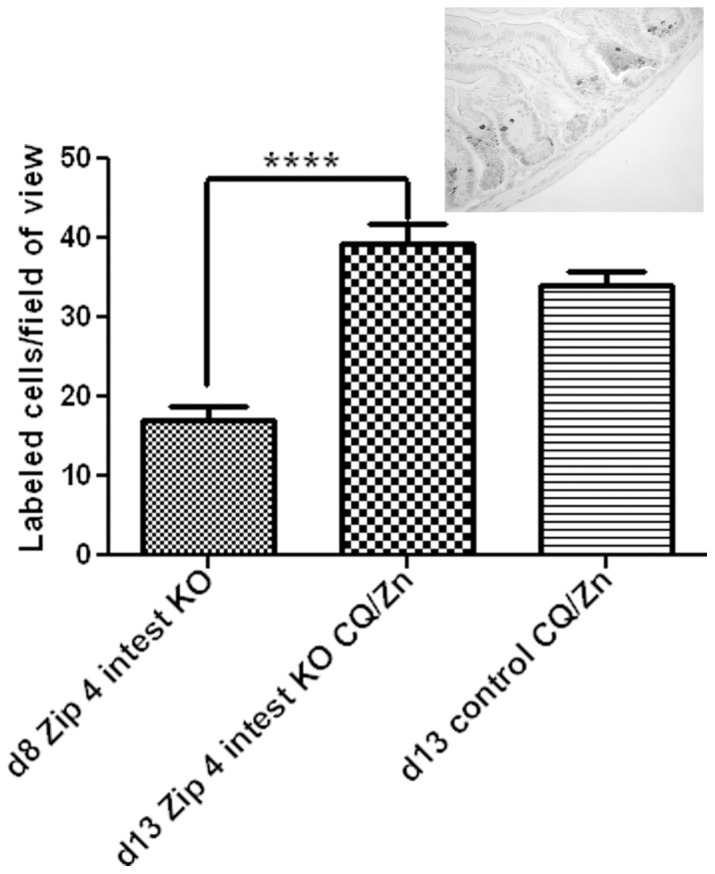
Quantification of phosphorylated-histone H3 labeled cells in sections of small intestine from *Zip4* ^intest KO^ mice before and after feeding CQ/Zn. *Zip4* ^intest KO^ mice and control mice (3 mice per group) were harvested on the indicated day after initiation of the knockout. Mice were fed chow containing CQ and provided with water containing zinc **(CQ/Zn)** on d8 and harvested on d13. The small intestines were fixed, paraffin embedded and sections were processed for IHC using an antibody against Ser 10 phosphorylated-histone H3. The number of positive cells per field of view was counted in multiple fields of view (n = 14) at 400X magnification in several sections from 3 mice and the values shown are the mean ± S.E.M. Statistical significance was determined using the Unpaired T-test (two-tailed). ****indicates P<0.0001. Sections from knockout mice fed CQ/Zn that had no detectable *Zip4* mRNA in the intestine were examined. **Insert**: A representative photomicrograph of the small intestine stained with antibody against phosphorylated-histone H3.

Loss-of-function of the intestine *Zip4* gene leads to repressed stem cell division in the intestine crypts [26]. The number of dividing cells in *Zip4* ^intest KO^ mice rescued with the CQ/Zn diet was quantified on d13 by counting the number of phosphorylated-histone H3 containing cells in sections of the small intestine (Fig. 4) from mice (n = 3) which did not contain detectable intestinal *Zip4* mRNA (See Fig. 3A). The results demonstrate that the rate of cell division in the crypts of the small intestine more than doubled by d13 in mice provided the CQ/Zn diet. The rate of cell division was restored to control levels (Fig. 4).

### Dietary CQ with Zinc Supplementation Restores Secretory and Absorptive Cell Differentiation

**Figure 5:**
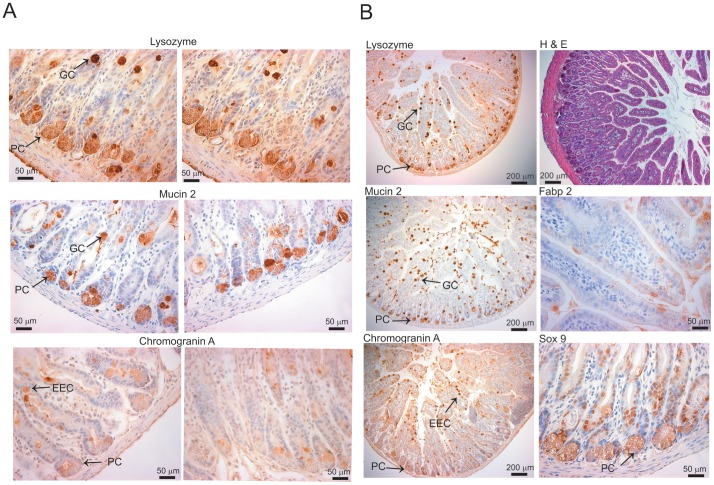
Development of the secretory and absorptive cell lineages in the small intestine is affected in *Zip4* ^intest KO^ mice. The small intestines from *Zip4* ^intest KO^ mice were harvested on d8 after initiation of the knockout. (**A**) Paraffin sections were processed for IHC using antibodies against antigens specific to the secretory cell lineages (**PC**: Paneth cell **lysozyme**, **GC**: Goblet cell **mucin 2** and **EEC**: Enteroendocrine cell **chromogranin A**). Brown color indicates positive immunostaining and the black arrows demarcate these cell types. (**B**) **Left hand panels** are lower magnification views of sections shown in (**A**). **Panels on the right** are paraffin sections stained with hematoxylin and eosin (**top right**), processed for IHC using an antibody against mouse fatty acid binding protein (**Fabp2**) which is enterocyte-specific (**middle right**) or antibody against mouse **Sox9** which is expressed in stem cells and Paneth cells (**lower right**). These results were reproduced in multiple sections from 4 *Zip4* ^intest KO^ mice (sections from 2 mice are shown), none of which had detectable *Zip4* mRNA in the intestine.

**Figure 6:**
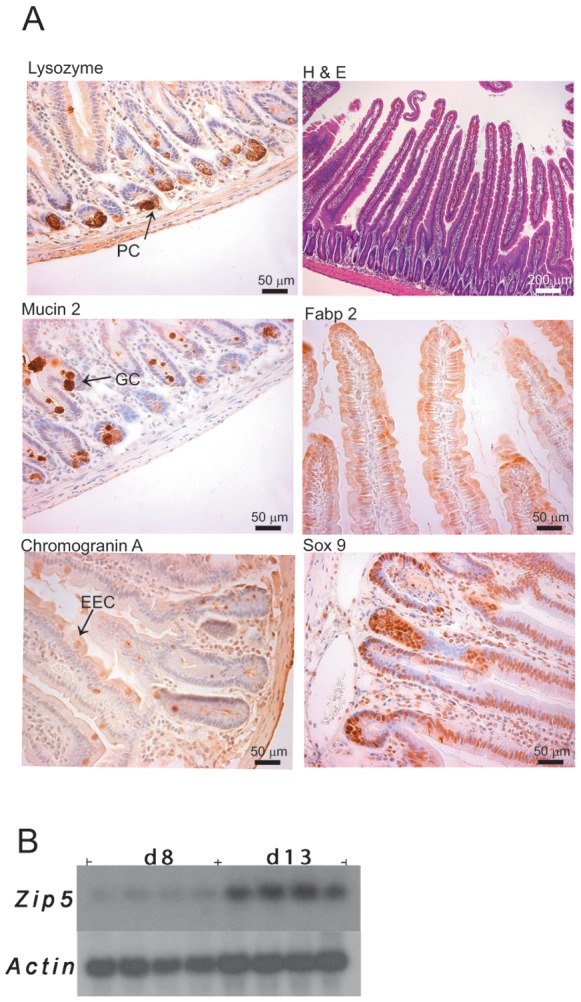
Proper development of the secretory and absorptive cell lineages in the small intestine is rapidly restored in *Zip4* ^intest KO^ mice fed CQ/Zn. The small intestines from *Zip4* ^intest KO^ mice were harvested on d13 after initiation of the knockout and starting CQ/Zn on d8. Sections from these mice which did not have detectable *Zip4* mRNA were examined. (**A: left hand panels**) Paraffin sections were processed for IHC using antibodies against antigens specific to the secretory cell lineages (**PC**: Paneth cell **lysozyme;** **GC**: Goblet cell **mucin 2;** and **EEC**: Enteroendocrine cell **chromogranin A**). Brown color indicates positive immunostaining. (**A: Right hand panels**) **P**araffin sections were stained with hematoxylin and eosin (**top right**), processed for IHC using an antibody against mouse fatty acid binding protein (**Fabp2**) which is enterocyte specific (**middle right**) or antibody against mouse **Sox9** which is expressed in stem cells and Paneth cells (**lower right**). These results were reproduced in multiple sections from 4 *Zip4* ^intest KO^ mice and the results were indistinguishable from those obtained using sections from control mice treated similarly. (**B**) Northern blot detection of *Zip5* mRNA in the intestine from *Zip4* ^intest KO^ mice on d8 and or d13 fed CQ/Zn. ZIP5 is a basolateral zinc transporter that is most abundant in the crypts but is also detectable on villus enterocytes [65].

Loss-of-function of the intestine *Zip4* gene leads to loss of intestine integrity and disruption of the stem cell niche by d6 after initiation of the knockout [26]. These findings were extended by examination of the small intestines on d8 (Fig. 5) and those on d13 given the CQ/Zn diet (Fig. 6). Results from the d13 knockout mice given the CQ/Zn diet that did not have detectable *Zip4* mRNA in the intestine are shown, but these results were indistinguishable from those found in similarly treated mice that were found to have intestinal *Zip4* transcripts. Immunohistochemical detection of proteins unique to the secretory cell lineage revealed a remarkable pattern on d8 (Fig. 5A). Lysozyme, which is normally restricted to the Paneth cells, was also abundant in cells dispersed throughout the villus epithelium (Fig. 5B) and mucin 2, which is normally restricted to goblet cells in the villus epithelium, was also readily detectable in Paneth cells at the base of the crypts (Fig. 5A & B). Furthermore, chromogranin A, which is normally restricted in the intestine to the enteroendocrine cells in the villus epithelium, was also detected in Paneth cells at the base of the crypts, though the staining is relatively weak (Fig. 5A & B). As shown previously [26] the pattern of expression of the transcription factor Sox9 (sex determining region Y-box 9) which plays an important role in stem cell proliferation and differentiation in the intestine is altered in the knockout mice. Nuclear staining is largely absent, Paneth cells show a diffuse cytoplasmic staining and many cells in the crypts do not appear to contain Sox9 on d8 (Fig. 5B). Thus, the differentiation of stem cell progeny which give rise to the secretory cell lineage is improper in *Zip4* ^intest KO^ mice. Immunohistochemical detection of fatty acid binding protein 2 (Fabp2), which is unique to the absorptive cell lineage, revealed very few positive cells on d8 and histological examination showed a disordered villus epithelium with few columnar epithelia cells and shortened villi indicative of abnormal development of the absorptive cell lineage in *Zip4* ^intest KO^ mice (Fig. 5B: Compare Fig. 5 with Fig.6).

The intestine from *Zip4* ^intest KO^ mice which were provided dietary CQ/Zn beginning on d8 was examined on d13 (Fig. 6). IHC revealed that the markers for the secretory lineages (lysozyme, mucin2 and chromogranin A) each displayed patterns of expression indistinguishable from those of control mice (Fig. 6A). Lysozyme was restricted to the Paneth cells, mucin 2 was abundant in goblet cells moving up into the villus epithelium and chromogranin A staining was restricted to enteroendocrine cells dispersed in the villus epithelium. Staining for Sox9 was intense in the nuclei of crypt cells. Examination of intestine histology revealed an intact columnar epithelium and villi and crypts which appeared to be normal, and fatty acid binding protein 2 staining was detectable in most of the enterocytes in the villus epithelium. In addition, the mRNA encoding the basolateral zinc transporter ZIP5 was increased significantly in relative abundance (Fig. 6B). Taken together the above results suggest that the CQ/Zn diet leads to rapid restoration of the integrity of the intestine within 5 days of starting the diet in these *Zip4* ^intest KO^ mice by ameliorating the effects of zinc deficiency on intestinal stem cell division and the differentiation of the stem cell’s progeny.

### Dietary CQ with Zinc Supplementation Restores Essential Metal Homeostasis

**Figure 7:**
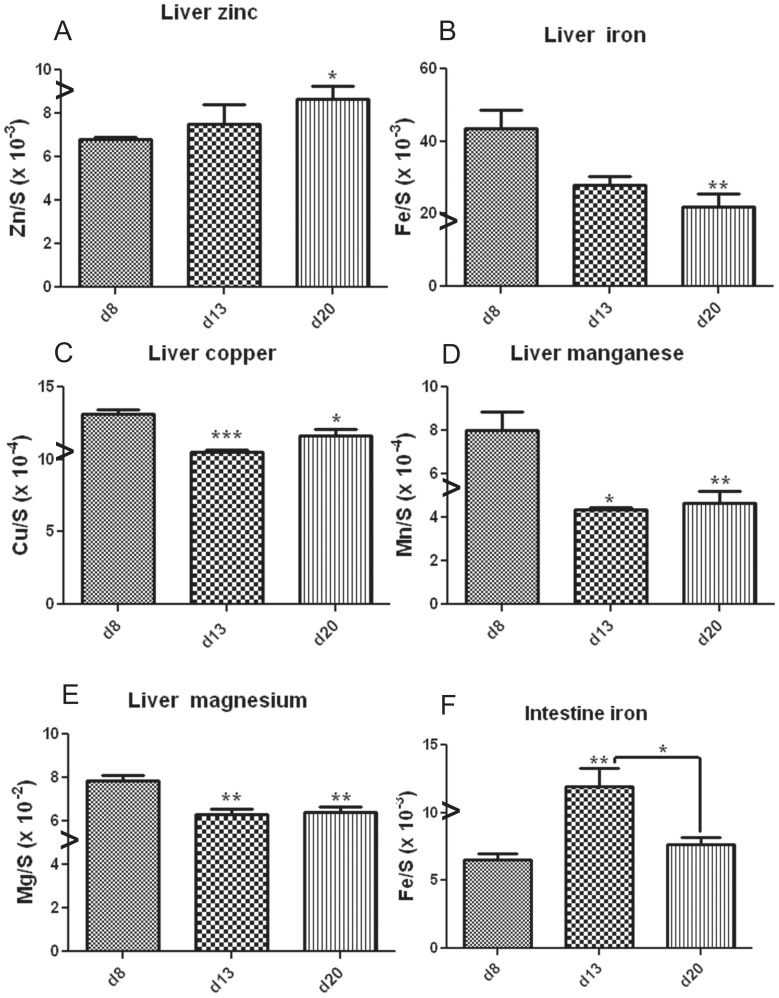
ICP-MS quantification of essential metals in *Zip4* ^intest KO^ mice before and after feeding CQ/Zn. The small intestine, liver and pancreas from *Zip4* ^intest KO^ mice were harvested on d8 after initiation of the knockout and on d13 and d20 after starting CQ/Zn on d8 (3 to 5 mice per group). Only data that showed significant changes are presented. Concentrations (ppm) for liver zinc (**A**), iron (**B**)**,** copper (**C**), manganese (**D**)**,** and magnesium (**E**)**,** and intestine iron (**F**)**,** were normalize to sulfur concentrations (ppm). Individual tissue samples were analyzed by ICP-MS for multiple elements including these essential metals. Data are expressed as metal/sulfur ratio ± S.E.M. Statistical significance was determined using the Unpaired T-test (two-tailed). Values were considered different if P<0.05. *indicates P<0.05; **indicates P<0.01; ***indicates P<0.001. Our previous studies showed that iron, copper and manganese accumulate in the liver when *Zip4* is knocked out in the intestine whereas iron is reduced in the intestine [26]. The symbol **(>)** on the Y-axes indicates approximate metal/sulfur ratios found in control mice.

Our previous studies of *Zip4* ^intest KO^ mice revealed that the homeostasis of several essential metals was progressively perturbed after initiation of the knockout. Elemental and essential metal analyses of small intestine, liver and pancreas showed that iron, manganese and copper accumulated to high levels by d8 in the liver [26]. Elemental and essential metal analyses were performed using inductively coupled plasma mass spectrometry (ICP-MS) of the above organs harvested from d8 *Zip4* ^intest KO^ mice and from d13 and d20 mice that had been provided dietary CQ/Zn (Fig. 7). Only changes that were statistically significant are shown and essential metal concentrations were normalized to sulfur concentrations as an internal control. Liver zinc increased after beginning the CQ/Zn diet, consistent with the concept that this ionophore is bringing zinc into the mice. Zinc levels were variable between individual mice but on d20, a statistically significant increase of zinc (28%) was noted in the liver (Fig. 7A). In contrast, decreased concentrations of iron (36%), copper (20%) and manganese (45%) in the liver were found (Fig. 7B-D). It was also noted that magnesium concentrations in the liver decreased 20% (Fig. 7E) by d13 and, in addition, manganese concentration in the pancreas transiently decreased (36%) on d13 but rebounded by d20 (data not shown). Our previous studies showed that the small intestine had lost ∼50% of its iron by d4 after the knockout was initiated [26]. Herein it was found that intestine iron increased 81% by d13 in mice provided the CQ/Zn diet and then declined (Fig. 7F). Taken together, these results suggest that the homeostasis of several essential metals is restored after providing extremely zinc deficient *Zip4* ^intest KO^ mice with dietary CQ/Zn.

## Discussion

We utilized a mouse model of the lethal human genetic disease acrodermatitis enteropathica as a system in which to examine approaches toward treating zinc deficiency, a world-wide health issue. We found that the ionophore CQ and zinc supplementation acted synergistically to ameliorate extreme zinc deficiency, leading to restored growth and preventing lethality. These effects involved stimulating the intestine stem cells to divide and their progeny to differentiate thus repairing the structure and function of the small intestine. Previous studies have shown that zinc can stimulate DNA synthesis in embryonic stem cells [39] and that zinc deficiency can impair the proliferation of adult neuronal stem cells [40]. Further, the expression of *Zip4* can repress apoptosis, enhance cell cycle and increase migration in hepatocellular carcinoma and pancreatic cancer [41]–[43] and over-expression of the zinc transporter *Zip1* can induce the osteogenic differentiation of mesenchymal cells [44]. Thus, zinc serves fundamentally important roles in many aspects of development and differentiation. The results reported are the first to show an *in vivo* function of zinc (with CQ) to stimulate proliferation of intestine stem cells and differentiation of their progeny. The associated recovery of rapid growth of CQ/Zn-treated *Zip4 ^intest KO^* mice suggests that the progenitor cells in many organs were stimulated to divide and differentiate. This may reflect the reestablishment of the ability of the intestine to take up many nutrients in addition to the increased influx of zinc. But, many recent studies have documented the effects of zinc on important signal transduction cascades [45], [46]. Extracellular zinc can stimulate epithelia repair via binding to the zinc receptor [47] and the zinc and iron transporter ZIP14 can modulate c-Met activity during liver regeneration [48]. Fluctuations in intracellular zinc can modulate IGF-1 signaling [49] and ZIP7 may function to release intracellular stores of zinc and thereby modulate tyrosine kinase activity [50]. Thus, there is a wealth of data emerging on the mechanisms of action of zinc. However, high dose zinc supplementation has recently been reported to induce memory impairment in mice after a three month exposure to 60 ppm zinc in drinking water [17] and excess zinc can impair the uptake of copper and iron with potentially devastating effects [14], [16]. Therefore, zinc supplementation must be tightly controlled. Perhaps by increasing the efficacy of zinc uptake by functioning as an ionophore, CQ could allow for the administration of safer dosages of zinc supplementation that avoid toxic effects. Some patients fail to thrive with high-dose zinc supplementation [28] or with CQ treatment alone [31] and they might benefit most by oral CQ with zinc supplementation.

In contrast to zinc, the mechanisms of action of CQ are not as well understood. In the studies reported herein the ability of CQ to act as a zinc ionophore was of primary importance to its efficacy [51]. However CQ may also have helped to reduce or prevent infections in the intestine of these knockout mice given its activity against intestinal diseases [30]. A recent screen in yeast identified 8 different 8-hydroxyquinolines that could synergistically rescue toxicity of proteins associated with neurodegenerative diseases which suggests that each compound exerted a different function [38]. It is therefore possible that alternate 8-hydroxyquinolines may be found that are more effective than CQ at reversing the effects of zinc deficiency. The therapeutic agent PBT2 is an example of an ionophore in this class of compounds which is in clinical trial for treatment of Alzheimer’s disease [52], [53] and has been shown to promote the degradation of Aβ while enhancing the phosphorylation of GSK3 kinase apparently via inhibition of the phosphatase calcineurin [54]. Radiolabeled CQ in APP mice is thought to be complexed with metallated Aβ in the brain and to attenuate accumulation of Aβ in Tg2576 mice [55], [56]. Recent studies of PBT2 suggested improvement in cognition [29], [30], [57]. Thus, 8-hydroxyquinolines may affect signal transduction cascades independent of their functions as ionophores.

The dosage and route of administration of CQ and of zinc supplementation are important considerations when treating zinc deficiency. CQ has been reported to reduce the viability of cancer cell lines in culture perhaps by chelating intracellular zinc and copper [58]. Therefore, CQ in the absence of exogenous zinc could be toxic. The toxicity of zinc is also enhanced by CQ. A CQ/zinc chelate was reported to be cytotoxic to melanoma cells in culture and to target zinc to lysosomes [59], [60] but the cells were exposed to high concentrations of zinc in the presence of CQ and it is perhaps not surprising that cytotoxicity occurred in those experiments. Intraperitoneal (I.P.) injections of CQ were shown to inhibit xenograph tumor growth in nude mice [58] and dietary CQ in feed has been reported to increase lethality in amyloid precursor protein transgenic mice after several months but the effect was modest and was prevented by the inclusion of supplemental copper [37]. Clearly CQ and zinc can both be toxic depending on dosage and concentration. The route of administration of CQ is also important to consider. CQ injected I.P. causes a dramatic reduction of zinc in brain, testes and pancreas in mice whereas oral CQ does not [61]. Plasma levels of CQ are much higher after I.P. injection than after oral ingestion and 12% bioavailability has been reported in hamsters (Reviewed by [30]). CQ increases zinc absorption in rats and humans in a dose dependent manner [62].

During the course of our experiments we noted that ∼20% of *Zip4 ^intest KO^* mice given dietary CQ/Zn failed to thrive and ∼56% of those appeared to be blind in one or both eyes. This apparent blindness is highly reminiscent of the optic atrophy that developed in some AE patients who were treated with diiodohydroxyquinoline [34] and of the SMON syndrome found in some Japanese who were taking CQ or diiodohydroxyquinoline. We noted that this syndrome was exacerbated in *Zip4 ^intest KO^* mice while being fed dietary CQ if they could not easily access the supplemental zinc in the drinking water due to their small size and deteriorated physical condition. In addition, it only occurred in those individuals that failed to thrive when given dietary CQ/Zn. This problem was not found in control mice fed the CQ/Zn diet. We previously reported that mice heterozygous for *Zip4* ^+/−^are hypersensitive to zinc deficiency and about 22% of the heterozygous offspring at weaning failed to thrive and actually lacked one or both eyes [63]. Taken together these studies support the notion that inadequate zinc intake coupled with dietary CQ can be dangerous and that zinc deficiency is the underlying cause of blindness in our studies. We further speculate that maternal zinc deficiency during pregnancy may predispose some individuals to SMON since we know that maternal heterozygosity of *Zip4* ^+/−^ predisposes her heterozygous offspring to optic atrophy in the mouse model [63]. Future dose-response studies in this mouse model system might help to determine if there is an optimal concentration of CQ and zinc supplementation that can rescue all of these *Zip4 ^intest KO^* mice or if there is a different 8- hydroxyquinoline derivative that might be more effective.

## Materials and Methods

### Animals and Ethics Statement

Experiments involving mice were performed in accordance with the guidelines from the National Institutes of Health for the care and use of animals and were approved by the University of Kansas Medical Center Institutional Animal Care and Use Committee (Protocol #2012–2057). Acrodermatitis enteropathica is a lethal disease in humans as it is in this mouse model, and due to variability among animals in their response to loss-of-function of intestine ZIP4, it was not possible to avoid the death without humane intervention of a small number of the mice used in these experiments. This unfortunate consequence is justified given the potential for these studies to help in the treatment of devastating human diseases of zinc deficiency. However, mice were monitored at least daily or often times twice daily and every effort was made to humanely euthanize the vast majority of mice by cervical dislocation as soon as they appeared listless and apparently unable to eat due to the effects if this disease. The maximum weight loss by individual mice before humane intervention in these experiments ranged between 25% and 30%. Mice were maintained on normal mouse chow with free access to deionized water and where indicated were provided with drinking water containing 250 ppm ZnSO_4_ and/or chow containing 250 ppm clioquinol. Zinc containing water was changed every two days. This dosage of zinc equates to ∼75 mg zinc/kg body weight/day and ∼50 mg CQ/kg body weight/day. Clioquinol was purchased from Santa Cruz Biotechnology and chow containing CQ was prepared by Dyets Inc.

### Conditional Deletion of the Intestine Zip4 (Slc39a4) Gene

We previously described the structure of the floxed mouse *Zip4* (Slc39a4) gene and the creation of mice homozygous for this targeted floxed gene [26]. These mice were crossed with transgenic mice bearing a tamoxifen-dependent Cre recombinase (*vil-Cre-ERT2*) expressed under the control of the *villin* promoter to allow for inducible deletion of the *Zip4* gene specifically in the intestine [64]. Mice heterozygous for *vil-Cre-ERT2* and homozygous *Zip4^FX/FX^* were crossed with *Zip4^FX/FX^* mice to yield 50% offspring with *Zip4^FX/FX^*: *vil-Cre-ERT2* alleles and 50% with *Zip4^FX/FX^* alleles. The latter provided age and genetically matched controls for our experiments and were labeled as control (con) in the figures. Deletion of the intestine *Zip4* gene was initiated by the I.P. injection of 100 µl of tamoxifen (1 mg) daily for 3 consecutive days beginning 2 to 3 weeks post-weaning. These are referred to as *Zip4* ^intest KO^ mice throughout the manuscript.

### Northern Blot Hybridization

Total RNA was isolated using Trizol reagent (Invitrogen). Total RNA (3–6 µg) was size-fractionated by agarose-formaldehyde gel electrophoresis, transferred and UV cross-linked to a Zeta Probe GT nylon membrane (BioRad). Northern blot membranes were prehybridized, hybridized then washed as described in detail previously [26]. Hybrids were detected by autoradiography at −80°C. Duplicate gels were stained with acridine orange to ensure equivalent loading and integrity of total RNA. Riboprobes for mouse *Actin, Zip4*, and *Zip5* [65] were described previously. Probes were used at 2×10^6^ cpm/ml of hybridization solution.

### Antisera

The following antibodies were used at the indicated dilution for immunohistochemistry (IHC): Sox9 (Millipore: 1∶750); lysozyme (DakoCytomation: 1∶150); phosphor (Ser10)-histone H3, (Cell Signaling: 1∶100); mucin 2 (Santa Cruz Biotechnology: 1∶200); fatty acid binding protein 2 (Fabp2: OriGene: 1∶300); and chromogranin A (Research Diagnostics, Inc.: 1∶100).

### Immunohistochemistry (IHC)

The proximal small intestine was collected, flushed with cold PBS, cut into small pieces and fixed in Bouin’s fixative or 4% paraformaldehyde in PBS overnight at 4°C. Fixed tissues were embedded in paraffin and sections (1 µm) were prepared by Histo-Scientific Research Laboratories (HSRL). Bouin’s fixed sections were deparaffinized, rehydrated and stained with hematoxylin-eosin for examination of gross morphology. For IHC, paraformaldehyde fixed serial sections were deparaffinized, rehydrated and antigens were retrieved in 10 mM citrate, pH 6.0, using a 2100 Retriever pressure cooker (PickCell Laboratories) or using proteinase K digestion (20 µg/ml in 50 mM Tris base, pH 8.0, 5 mM EDTA) at 37°C for 10 min for the lysozyme and chromogranin A antibodies. Samples were processed using the Histostain Plus LAB SA Detection System (Invitrogen) for rabbit antibodies or with the VectorStain ABC anti-goat kit (Vector Labs) for the chromogranin A antibody according to the manufacturer’s instructions. Stained slides were counterstained briefly in Mayer’s hematoxylin (Sigma) and photographed using a Leica DM 4000B microscope (Leica-microsystems) with Adobe Photoshop image capture software (Adobe).

### Elemental and Essential Metal Determination

Elemental profiling via inductively coupled plasma mass spectrometry (ICP-MS) was performed at the Donald Danforth Plant Science Center Ionomics Core Facility in St. Louis, Missouri. The following elements were measured: Na, S, P, K, Ca, Fe, Co, Cu, Zn, Mn, Mg, As, Se, Mo and Cd. Mouse tissues (n = 3 to 5) were dried at 95°C in a vacuum oven and shipped to the Ionomics Core Facility for processing [66]. Element concentrations were determined as µg g^−1^ dry weight (ppm) of each element and were normalized to ppm S.

### Statistical Analyses

Graphs were generated and statistical analyses performed using GraphPad Prism5 software (GraphPad Software). Statistical significance was determined using the Unpaired T-test (two-tailed) and values were considered different if P<0.05. Data are expressed as the mean ± S.E.M. *indicates P<0.05; **, p<0.01; ***indicates P<0.001; ****indicates P<0.0001.
